# Determinants of medication adherence and knowledge among patients with Type 2 diabetes mellitus: A cross-sectional study in Northwestern China

**DOI:** 10.1371/journal.pone.0343091

**Published:** 2026-02-20

**Authors:** Jingjing Pan, Xiaorong Xue, Haiyan Li, Lian Wu

**Affiliations:** 1 Department of Pharmacy, Xi’an People’s Hospital (Xi’an Fourth Hospital), Xi’an, People’s Republic of China; 2 Department of Ophthalmology, Xi’an People’s Hospital (Xi’an Fourth Hospital), Xi’an, People’s Republic of China; Population Council Ethiopia, ETHIOPIA

## Abstract

**Objectives:**

The study aimed to evaluate medication knowledge and adherence among patients with type 2 diabetes mellitus (T2DM) in northwestern China, identify factors influencing adherence, and examine the role of medication knowledge in adherence behavior.

**Methods:**

This hospital-based,cross-sectional study was conducted at Xi’an People’s Hospital (Xi’an Fourth Hospital) from February to May 2025. A total of 510 adult patients with T2DM were enrolled. Medication knowledge and adherence were assessed using “diabetes medication knowledge questionnaire (DMKQ)” and “general medication adherence scale (GMAS)”, respectively. Multiple linear regression analysis was employed to identify independent risk factors associated with adherence.

**Results:**

The study revealed that 53.93% of patients achieved satisfactory medication adherence, while 58.04% possessed adequate medication knowledge. Forgetfulness or difficulty remembering medication schedules was the most frequently reported barrier to adherence. Notably, a substantial proportion of patients were unaware of potential medication side effects, and nearly half lacked sufficient knowledge on managing adverse effects. Multiple linear regression analysis identified gender (B = 0.564, *P* = 0.039), education level (B = 0.565, *P* = 0.032), living conditions (B = 0.776, *P* = 0.004), duration of antidiabetic drugs used (B = −0.046, *P* = 0.009) and medication knowledge (B = 1.318, *P* < 0.001) as independent factors of medication adherence, with medication knowledge exhibiting the strongest association (Beta = 0.533). Furthermore, a significant positive correlation was observed between medication knowledge and adherence.

**Conclusion:**

Patients with diabetes in northwestern China often demonstrate suboptimal levels of both medication knowledge and adherence. As medication knowledge positively affects medication adherence, healthcare providers should assume a more proactive role in delivering comprehensive medication education to this patient population.

## Introduction

Diabetes mellitus (DM) is a metabolic disease in which the pancreas does not secrete enough insulin, and/or the body develops resistance to insulin it produces [1]. It is characterized by organ dysfunction that arises directly or indirectly from the effects of chronic hyperglycemia [2]. As the disease progresses, patients are at risks of experiencing damages to the cardiovascular system, eyes, kidneys, neurons, and feet [3–5]. T2DM is the most common form of the disease, accounting for over 90% of all diabetes cases. In 2021, the global prevalence of T2DM among adults aged 20–79 years was 536.6 million people (10.5%), and this number is projected to rise to 783.2 million (12.2%) by 2045 [6]. China has witnessed the dramatic rises in diabetes prevalence over the past few decades, and it accounts for the largest number of people with diabetes(114million),11% of the adult population [7,8]. Diabetes not only affects individuals’ functional capacities and quality of life but also increases health expenditures [9]. The increasing prevalence of diabetes and its complications remains a huge burden globally [10].

The prevalence of diabetes continues to grow rapidly, yet it remains inadequately controlled. A study conducted in Latin America reported that the glycemic control rate among T2DM patients was 46.2% [11]. A multi-center survey conducted in China reported that the glycemic control rate was only 32.6% in Chinese T2DM outpatients [12]. Poor adherence to treatment regimens is a key contributor to uncontrolled glycemia [13]. Treatment adherence refers to the degree to which a person’s behavior-including medication intake, dietary compliance, and lifestyle modifications-align with the agreed upon recommendations from healthcare providers [14]. A previous study demonstrated that a 10% decrease in adherence was associated with a 0.14% increase in glycosylated hemoglobin [15]. Optimal diabetes treatment adherence has significantly positive association with glycemic control [16].

Barriers to adherence consist of multiple factors including the communication pattern between the patient and the health professional, the patient’s cognitive, the patient’s resources including financial, psychological, social support, and the patient’s knowledge [17,18]. Some of the barriers are under the patient’s control such as patient’s knowledge about the medicine. Patients’ knowledge of their medications encompasses all essential information needed for proper medication use. This comprehensive understanding includes the therapeutic objectives, correct dosage, appropriate administration times, safety precautions, as well as potential drug interactions and adverse reactions [19,20]. Some studies have shown that a solid understanding of medications is closely associated with enhanced medication adherence and more favorable outcomes in pharmacotherapy [21,22]. Therefore, providing medication knowledge education to patients is necessary to enhance their mastery of medication knowledge, thereby further improving treatment-related attitudes, promoting health behavior changes,and enhancing medication adherence [23].

Despite considerable research on medication adherence, significant geographical disparities remain in adherence to T2DM treatment. Our research was conducted at a tertiary hospital in Xi’an, the capital city of Shaanxi Province in northwestern China. There are significant regional economic disparities within China, and cities in the northwest, such as Xi’an, are generally less developed compared to those in the eastern and southern regions. Socioeconomic and culture factors are known to be important determinants of medication adherence. However, there is limited attention and reports on medication knowledge and adherence among diabetic patients in northwestern China. The present study aims to investigate the current status of medication knowledge and adherence,as well as the factors influencing adherence among diabetic patients in Xi’an-a major city in northwestern China- with a particular focus on the role of medication knowledge in shaping adherence behavior.

## Methods

### Study area

This study was a hospital-based, cross-sectional survey conducted at Xi’an People’s Hospital (Xi’an Fourth Hospital) in Xi’an, China, from February to May 2025. As the capital of Shaanxi Province in northwestern China, Xi’an serves as the region’s principal medical hub, hosting 58% of the province’s tertiary hospitals. Xi’an People’s Hospital (Xi’an Fourth Hospital) is a major tertiary Grade A hospital with 1,800 inpatient beds. In 2024, it recorded 2,469,774 outpatient visits and discharged 157,474 inpatients. The patients admitted to the hospital are mainly from Xi’an and its surrounding areas, and also include those from other regions within the province and some other provinces in the northwest region.

### Study population

Patients were eligible for inclusion if they met the following inclusion criteria: (1) aged 18 years or older; (2) diagnosed with T2DM; (3) undergoing antidiabetic oral drug therapy, and (4) willing to participate in the study. Exclusion criteria were as follows:(1) suffering from severe or terminal diseases, (2) unable to communicate due to physical or mental impairment; and (3) being pregnant.

The minimum sample size was calculated using the following formula: s = z^2^[p(1-p)]/d^2^ [24], where s is the minimum sample size, z refers to the standard normal variate, p represents an estimate of treatment adherence and d is the sample error to be tolerated. The confidence interval is 5%; therefore, d is 0.05, z is 1.96, and p was 59% from previous study [25]. The calculated minimum sample size was 371 participants. A total of 510 eligible patients agreed to participate and were ultimately enrolled in the study, all of whom completed the survey.

### Data collection procedures and tools

Patient sociodemographic data including age, gender, and occupational status were obtained from electronic medical records. Education level, living conditions and clinical data including duration of antihyperglycemic drugs used and the number of antihyperglycemic drugs used were collected through interviews conducted by trained clinical pharmacists when not available in the electronic medical records.

Medication adherence was measured using the Chinese version of the general medication adherence scale (GMAS). The scale was developed by Naqvi et al. and was translated to Chinese version [26]. The Chinese version of the GMAS scale had been proved to be a valid and reliable instrument to identify the levels of medication adherence and possible barriers for adherence of the medication regime for patients with chronic diseases [27]. This scale includes 3 dimensions and 11 items. Each response ranges from 0 to 3 on Likert scale: always-0 point, mostly-1 point, sometimes-2 points, never-3 points. Item scores are summed to produce an overall adherence score of 0–33, with higher scores indicating better adherence. Scores of 30–33 points indicated high adherence, 27–29 points represented good adherence, 17–26 points reflected partial adherence, 11–16 points denoted low adherence, and 0–10 points signified poor adherence.High adherence and good adherence were classified as satisfactory adherence.

Patient knowledge regarding antidiabetic medications was evaluated using a questionnaire developed by McPherson et al, which was based on the five key medication issues patients should understand for any prescription medication [28]. The diabetes medication knowledge questionnaire (DMKQ) was specifically designed to evaluate knowledge about a diabetes medication, rather than knowledge of the disease itself. The questionnaire comprises seven validated yes/no questions. The total medication knowledge score is calculated based on the number of accurate responses, with one point awarded for each correct answer and zero points for incorrect or unanswered items. Notably, an additional point is assigned to participants who correctly identify the precise mechanism of action of their medications in response to question 2. Consequently, the scoring range for this section spans from 0 to 8, with a score of ≥5 indicative of a good knowledge of antidiabetic drug [20,29]. In the present study, we used the Chinese version of the DMKQ. The original English questionnaire was translated into Chinese and back-translated into English to ensure conceptual equivalence in this study. A pilot study involving 30 patients with diabetes was carried out to confirm the comprehensibility of the Chinese version. All participants reported no difficulty in understanding or responding to any of the items.

### Data analysis

Descriptive statistics were used to analyze sociodemographic data, clinical characteristics, medication knowledge, and adherence status among T2DM patients. Categorical variables are presented as frequencies and percentages (%), while continuous variables are summarized as mean ± standard deviation. Multiple linear regression analysis was employed to identify independent risk factors associated with medication adherence. Statistical significance was defined as *P* < 0.05. All analyses were performed using SPSS version 19 (IBM Corp., Armonk, NY, USA).

### Ethical approval

The study was approved by the Ethics Committee of Xi’an People’s Hospital (Xi’an Fourth Hospital). All participants provided written informed consent after receiving a detailed explanation of the study’s objectives and procedures. The research strictly adhered to the ethical principles of the Declaration of Helsinki and complied with applicable Chinese clinical research regulations. Prior to questionnaire administration, investigators thoroughly explained the study’s purpose and provided standardized instructions to participants. Patients were instructed to complete the questionnaires independently. For those unable to read Chinese, investigators assisted by reading each item to facilitate questionnaire completion.

## Results

Of the 510 participants, 305 (59.80%) were male, and 212 (41.56%) of them were above 65 years old. 190 (37.25%) participants were retired and those with a high school education or below accounted for 64.51%.A total of 23 participants (4.51%) lived alone, 367 participants (71.96%) lived with their spouses, and 120 participants (23.53%) lived with their children. Approximately 29.02% of the participants had been prescribed oral antidiabetic drugs for less than five years, while 18.63% had been taking them for over twenty years. Additionally, 218 participants (42.75%) took three or more oral antidiabetic drugs daily (Table 1).

**Table 1:** Sociodemographic and Clinical Characteristics of T2DM Patients (n = 510).

Characteristics	Frequency(n)	Percentage(%)
Gender		
Male	305	59.8
Female	205	40.2
Age		
< 45	52	10.2
45-64	246	48.24
65-79	185	36.27
≥ 80	27	5.29
Occupation		
Unemployed	191	37.45
Employed	129	25.29
Retired	190	37.25
Education level		
Primary school or below	100	19.61
High school	229	44.9
College/University	181	35.49
Living condition		
Live alone	23	4.51
Live with spouse	367	71.96
Live with children	120	23.53
Duration of diabetes drugs used (years)		
< 5	148	29.02
05-Sep	95	18.63
Oct-19	172	33.73
≥ 20	95	18.63
Number of diabetes drugs used		
1	105	20.59
2	187	36.67
≥ 3	218	42.75

The study findings revealed that the mean GMAS score was 26.49 ± 3.44. 53.93% of the participants demonstrated satisfactory medication adherence, comprising 19.22% with high adherence and 34.71% with good adherence. The remaining 46.07% of subjects exhibited unsatisfactory adherence, distributed as follows: 45.29% showed partial adherence, 0.59% had low adherence, and 0.2% demonstrated relatively poor adherence. Regarding diabetes medication knowledge, the mean score was 4.89 ± 1.39. Over half of the participants(58.04%) demonstrated satisfactory knowledge, while 41.96% exhibited low knowledge (Table 2).

**Table 2:** Descriptive statistics for the general medication adherence scale (GMAS) and diabetes medication knowledge questionnaire (DMKQ) (n = 510).

Variables	N(%)
**GMAS Score(mean±SD)**	26.49 ± 3.44
Level of adherence	
High adherence score (30–33)	98 (19.22%)
Good adherence score (27–29)	177 (34.71%)
Partial adherence score (17–26)	231 (45.29%)
Low adherence score (11–16)	3 (0.59%)
Poor adherence score (0–10)	1 (0.20%)
**DMKQ Score(mean±SD)**	4.89 ± 1.39
Level of medication knowledge	
Satisfactory knowledge (score≥5)	296 (58.04%)
Low knowledge (score<5)	214 (41.96%)

The participants’ scores of DMKQ for each question were showed in Table 3. It can be seen that correct response rates for questions 5 and 6 were relatively low, particularly for question 5, where only 21.57% of participants answered correctly. The overwhelming majority of patients were unaware of the possible side effects from their medications and nearly half lacked adequate knowledge about how to handle side effects when they occurred. In addition, only 64.51% participants were able to list all the names of the medications they were taking.

**Table 3:** Distribution of items in the diabetes medication knowledge questionnaire (DMKQ) of T2DM patients (n = 510).

Items of DMKQ scale	Score(M ± SD)	Frequency (Yes)	Percentage(%)
Q1. Can you list the names of all medications you are currently taking?	0.65 ± 0.48	329	64.51
Q2. Can you tell me why you are taking this medication?	0.92 ± 0.40	448	87.84
Q3. Do you know how to take your medicine?	0.93 ± 0.26	472	92.55
Q4. Do you know when to take your medicine?	0.87 ± 0.34	443	86.86
Q5. Do you know the possible side effects of your medicine?	0.22 ± 0.41	110	21.57
Q6. Do you know what to do if your medication’s side effects occur?	0.52 ± 0.50	264	51.76
Q7. Do you know what to do if you miss a dose of your medicine?	0.80 ± 0.40	407	79.80

The average scores according to the GMAS, as well as the percentage and distribution for each result of medication adherence in each item, were presented in Table 4. It was found that difficulty remembering to take medication or forgetfulness was the main obstacle to adherence. Question 1 “Do you have difficulty in remembering to take your medications?”, question 2 “Do you forget to take your medication due to your busy schedule” and question 7 “Do you find it is a hassle to remember your medications due to medication regime complexity?” in the questionnaire had the fewest respondents who choose “never”, and their scores were relatively low, being 1.95 ± 0.77, 2.01 ± 0.61 and 1.96 ± 0.89, respectively.

**Table 4:** Distribution of items in the general medication adherence scale (GMAS) of T2DM patients (n = 510).

Items of GMAS	M ± SD	Always,n(%)	Mostly,n(%)	Sometimes,n(%)	Never,n(%)
**Dimension 1:Non-adherence due to patient behavior (un-intentional and intentional)**					
Q1. Do you have difficulty in remembering to take your medications?	1.95 ± 0.77	20(3.92%)	105(20.59%)	267(52.35%)	119(23.33%)
Q2. Do you forget to take your medication due to your busy schedule, travelling, meeting, events at home, party, marriage, religious celebrations, etc.?	2.01 ± 0.61	8(1.57%)	67(13.14%)	348(68.24%)	88(17.25%)
Q3. Do you discontinue your medication when you feel well?	2.52 ± 0.62	7(1.37%)	12(2.35%)	198(38.82%)	294(57.65%)
Q4. Do you stop taking medications when you feel adverse effects such as gastric discomfort, etc.?	2.56 ± 0.57	1(0.20%)	15(2.94%)	191(37.45%)	302(59.22%)
Q5. Do you stop taking medications without informing the doctor?	2.46 ± 0.58	2(0.39%)	15(2.94%)	237(46.47%)	256(50.20%)
**Dimension 2:Non-adherence due to additional disease and pill burden**					
Q6. Do you discontinue your medicines due to other medicines that you have to take for your additional disease?	2.73 ± 0.49	2(0.39%)	6(1.18%)	118(23.14%)	384(75.29%)
Q7. Do you find it is a hassle to remember your medications due to medication regime complexity?	1.96 ± 0.89	26(5.10%)	136(26.67%)	181(35.49%)	167(32.75%)
Q8. During the last month, had there been any occasion when you missed your medicines due to progression of disease and addition of new medicines?	2.64 ± 0.56	3(0.59%)	11(2.16%)	151(29.61%)	345(67.65%)
Q9. Do you alter medication regimen, dose and frequency by yourself?	2.35 ± 0.62	6(1.18%)	23(4.51%)	269(52.75%)	212(41.57%)
**Dimension 3:Non-adherence due to financial constraints**					
Q10. Do you discontinue these medications because they are not worth of the money you spent on them?	2.83 ± 0.42	3(0.59%)	5(0.98%)	71(13.92%)	433(84.90%)
Q11. Do you find it difficult to buy your medicines because they are expensive?	2.48 ± 0.77	13(2.55%)	49(9.61%)	131(25.69%)	318(62.35%)

Five factors were found to have an independent association with diabetes medication adherence: gender, education level, living condition, duration of antidiabetic drugs used and medication knowledge. Males (B = 0.564, *P* = 0.039) were less adherent to their medication treatment than females. Medication adherence increased as the education level of the diabetes patients grew (B = 0.565, *P* = 0.032). Compared to those living alone, subjects residing with a spouse or children demonstrated progressively higher medication adherence (B = 0.776, *P* = 0.004). Medication adherence decreased with the prolonged duration of diabetes medication use (B = −0.046, *P* = 0.009). The study demonstrated a positive correlation between DMKQ and GMAS scores, indicating enhanced medication adherence concomitant with increased medication knowledge (B = 1.318, *P* < 0.001). Furthermore, among the five independent influencing factors of medication adherence, medication knowledge had the greatest impact on adherence (Beta = 0.533) (Table 5).

**Table 5:** Multiple linear regression analysis of factors associated with adherence in T2DM patients.

Variables	B	SE	Beta coefficient	*P*-value	VIF
**Gender**	0.564	0.273	0.081	**0.039**	1.103
Age	0.013	0.014	0.047	0.330	1.650
Occupation	−0.142	0.203	−0.036	0.483	1.885
**Education level**	0.565	0.263	0.119	**0.032**	1.088
**Living condition**	0.776	0.269	0.112	**0.004**	2.233
**Duration of diabetes drugs used (years)**	−0.046	0.018	−0.106	**0.009**	1.181
Number of diabetes drugs used	0.129	0.179	0.029	0.474	1.156
**Medication knowledge**	1.318	0.095	0.533	**<0.001**	1.077

**SE**:standard error.

## Discussion

The study was carried out to evaluate the medication knowledge and adherence regarding the antidiabetic medications among patients with T2DM in northwestern China. For diabetes patients, maintaining medication adherence is crucial as it not only enhances glycemic control but also reduces the risk of complications [30]. Similarly, a higher level of knowledge about diabetes and antidiabetic drugs is strongly correlated with more effective blood glucose management [31].

In this study, only 53.93% of participants demonstrated satisfactory medication adherence. This finding is consistent with previous research conducted in China involving a large cohort of 20,270 T2DM patients, which reported that 59.77% of patients had good medication adherence in 2019 [25]. The adherence rate observed in the present study is not only relatively low compared to developed countries such as the United States [32] but is also lower than rates reported in some African and Middle Eastern countries [33–35]. Although the differences in results may be attributed to variations in sampling design, sample size, study settings, or medication adherence scales used, the suboptimal medication adherence deserves greater attention from Chinese healthcare professionals and policymakers.

China has 114 million diabetic patients, placing a substantial and growing economic burden on the national healthcare system and public finances. In 2007, total health expenditure related to T2DM and its complications was estimated at USD 339 billion, accounting for 22.3% of China’s total health expenditure, and this figure is projected to nearly double by 2030 [36]. To address this, the country has implemented a tiered care model where tertiary hospitals collaborate with community health centers. These centers are primarily responsible for screening high-risk populations and providing long-term follow-up care [37]. At the same time, China’s national essential medicines policy has included key diabetes drugs in the insurance reimbursement list, significantly improving medication access for patients at the grassroots level [38]. However, diabetes patient education still heavily relies on physicians’verbal instructions. In busy clinical settings, physicians often have limited time for in-depth medication counseling. As a result, many patients do not fully understand their treatment plans, which negatively affects adherence and long-term health outcomes.

The most frequent forms of non-adherence in this study were difficulty remembering to take medications, forgetting due to busy schedules and challenges recalling doses because of complex medication regime. Previous studies identified other forms of non-adherence, including lack of time for prescription refills, concerns about side effects, or self-adjusting medication doses on physical conditions [39,40]. Patients with diabetes often take multiple medications simultaneously, and some are also accompanied by other chronic diseases, leading to numerous medications and complex treatment regimens. Polypharmacy with complex treatment plans can negatively affect medication adherence [41]. Physicians should consider this when prescribing multiple medications to patients. Moreover, the use of fixed-dose combination preparations can help reduce the number of medications taken, potentially improving medication adherence.

In this study, gender, education level, living conditions, duration of antidiabetic drugs used and medication knowledge were found to be associated with medication adherence. Female participants demonstrated higher adherence rates compared to males. This finding aligned with previous reports indicating that females exhibited better adherence to antidiabetic agents than males [42,43], though some studies contradicted this observation [33]. This disparity might be attributed to a combination of social, occupational, and behavioral factors. Socially, men might be more inclined to embrace health beliefs that downplayed the severity of illness or emphasize stoicism, resulting in deliberate non-adherence. Male participants were also reported experiencing frustration and stress when disclosing their diagnoses to others, alongside holding fatalistic attitudes toward disease management [44,45]. Occupationally, men in our sample might have been engaged in less flexible work environments (e.g., manual labor, driving) that disrupt medication schedules or access. Behaviorally, men typically showed fewer health-seeking behaviors and weaker daily medication routines, whereas women with diabetes tended to engage more actively in self-management and disease-related learning [46].

It was found that with the increase of education level, medication adherence also gradually increased. The results were consistent with a study that showed low education level as a crucial factor responsible for poor adherence to T2DM therapy [47]. Additionally, another previous study conducted on diabetic patients concluded that patients with college education had more self-care management on adherence than patients with low education levels [48]. However, a study conducted in Indonesian found that there was no relationship between education and adherence in diabetic patients [41]. The positive relationship between educational level and adherence in diabetes patients is relatively easy to understand. As educational level increased, patients may have a deeper understanding of diabetes-related knowledge. Their attention to health may also be enhanced, which could lead to higher adherence.

Living conditions also significantly influenced medication adherence. Patients residing with their children or spouses demonstrated higher levels of adherence compared to those living alone, resonating strongly with established theories of social support. It was showed that treatment adherence was found to have strong and positive correlation with their obtained social support in patients with chronic disease [49]. Living with a spouse or children provides tangible, emotional, and informational support [50–52]. Family members help with daily tasks such as chores, managing appointments, and care during illness, reducing daily stress and cognitive load [53]. They also offer emotional security by listening, validating feelings, and providing affection, which serves as a key protective factor for mental health [54]. Additionally, family often provides advice, health reminders, and helps with problem-solving [55,56]. In contrast, individuals living alone may face greater challenges in accessing consistent, reliable support.

It was found that the medication adherence decreased as the duration of taking hypoglycemic drugs increased in this study. A study conducted by Murwanashyaka et al reported the similar results, and it showed patients who took anti-diabetic drugs for 4–10 years and more than 10 years were more likely to experience non-adherence to medication when compared to those under anti-diabetic medications for less than 4 years [33]. Prolonged treatment may induce therapeutic fatigue in patients. In the initial stage of diagnosis, patients usually exhibit relatively high adherence due to fear of the disease and determination to control the condition actively. As time progresses, this sense of crisis diminishes, and their motivation to maintain strict self-discipline declines. Meanwhile, forgetfulness is also a common contributing factor. For elderly patients, age-related memory impairment may develop over time, leading to missed doses. Furthermore, long-term medication is often accompanied by disease progression, which may necessitate an increase in the types, dosages, or administration frequency of drugs. Such complex regimens significantly compromise medication adherence.

Our study focused on the impact of medication knowledge on medication adherence in diabetes patients. As for diabetes medication knowledge, the mean score was 4.89 ± 1.39. More than half of the participants (58.04%) had satisfactory knowledge with a score of ≥5. It was consistent with the study conducted by Khan et al, which used the same scale and reported a mean diabetes medication knowledge score of 4.8 ± 1.71 among patients [28]. In contrast, Muhammad Haskani et al. reported a significantly lower mean score of 3.37 ± 1.38 [20]. In this study, 78.43% of participants were unaware of potential side effects of their antidiabetic medications. Nearly half (48.24%) participants did not know how to manage these side effects. Such gaps in medication knowledge served as a barrier to medication adherence and can undermine treatment efficacy. Hypoglycemia is a common risk for individuals taking antidiabetic medications. It is crucial for patients to recognize this potential side effect to enable appropriate self-correction. Effective education from health providers plays a vital role in reducing hypoglycemia risk.

This study demonstrated that there was a positive correlation between medication knowledge and adherence among diabetic patients. It suggested that greater knowledge about diabetes medication was associated with improved adherence to prescribed treatments. This finding was in line with some previous studies [35,57]. However, a study conducted in Brunei Darussalam revealed that there was no significant relationship between diabetes knowledge, medication knowledge and medication adherence [20]. Limited knowledge about medication regimens may discourage medication adherence. Participants further reported that earlier education on the importance of disease control and knowledge about medications could provide clearer expectations regarding disease progression [58].

The educational interventions was reported to improve participants’ knowledge of their medication and the disease, as well as their medication adherence [59]. The findings of this study highlighted the importance of patient education, particularly in medication management. Healthcare providers such as doctors, clinical pharmacists, and nurses are responsible for providing patients with the knowledge about their disease and medication. Healthcare providers should deliver face-to-face education to inpatients and outpatients to enhance their awareness about the disease, treatment, risk of complications, thereby improving medication adherence. Additionally, to enhance patients’ comprehension of their medical conditions and treatment regimens, it is imperative to implement community – based initiatives. These could include organizing educational lectures on chronic diseases like diabetes and disseminating disease-related brochures. Furthermore, knowledge regarding diabetes fundamentals, healthy lifestyle and preventive measures should be regularly disseminated through diverse media channels including lectures, television programs, radio broadcasts and online platforms. Such efforts will not only empower patients with knowledge but also play a crucial role in promoting self – management and adherence to therapeutic plans.

The study has several limitations. First, the sample was drawn from a single regional hospital rather than employing a randomized, multi-regional sampling approach, and the majority of participants were local residents, which may limit the generalizability of our findings. Second, the assessment of medication knowledge and adherence relied on self-reported questionnaires, potentially introducing recall bias. Third, the relatively small sample size may have reduced the statistical power of the analysis. Fourth, the use of a consecutive sampling method may limit the representativeness of the sample and the generalizability of the findings. Last but not least, while the Chinese version of the Diabetes Medication Knowledge Questionnaire (DMKQ) has been validated for its applicability via content validity evaluation and preliminary data analysis, with an initial exploration of the association between diabetes medication knowledge and patient adherence, a comprehensive psychometric assessment of its reliability and validity remains unperformed owing to the constraints of sample size and study design. Future research should expand the sample size and further enhance the scientific rigor of this measurement tool through rigorous reliability and validity analyses.

## Conclusion

This study identified low medication adherence among patients with T2DM in northwestern China. Adherence was significantly associated with gender, education level, living conditions, duration of antidiabetic drugs used and medication knowledge. Notably, a positive correlation emerged between adherence and diabetes-specific medication knowledge. Given that nearly half of the patients demonstrated poor understanding of diabetes medications, we recommend targeted interventions to improve patient education on diabetes and pharmacotherapy. Healthcare providers should play an important role in enhancing adherence through education.

## Supporting information

S1 Data Supporting data for this study. It contains the raw data, statistical analysis results, and additional data provided in response to the reviewers’ comments. (XLSX)
